# Life-Cycle Assessment of Polypropylene Production in the Gulf Cooperation Council (GCC) Region

**DOI:** 10.3390/polym13213793

**Published:** 2021-11-02

**Authors:** Amzan Alsabri, Furqan Tahir, Sami G. Al-Ghamdi

**Affiliations:** Division of Sustainable Development, College of Science and Engineering, Hamad Bin Khalifa University, Qatar Foundation, Doha 34110, Qatar; amzan.alsabri@live.com (A.A.); futahir@hbku.edu.qa (F.T.)

**Keywords:** environmental impacts, LCA, life cycle assessment, petrochemical industry, polymers, polypropylene, PP

## Abstract

The environmental impacts of the polypropylene (PP) manufacturing process are not fully understood in the Gulf Cooperation Council (GCC) region. There is a growing interest in assessing the environmental impacts of this highly demanded product, especially for the petrochemical industry sector. This research examines the environmental impacts of the polypropylene manufacturing process using a life cycle assessment (LCA) approach. Gabi software is selected to carry out this research study and quantify the risks associated with manufacturing one ton of polypropylene, chosen as the functional unit for this LCA study. This work has the following merits: (i) an evaluation of environmental impacts specific to GCC region based on actual plant data; (ii) the results in this work can be used to evaluate LCA impacts of PP based products; and (iii) emphasizing the importance of waste management in reducing environmental impacts. This study shows that the polypropylene manufacturing process releases numerous pollutants into the environment, as the gross CO_2_ emissions for the manufacturing process of PP in the plant located in the GCC region were estimated to be 1.58 kg CO_2_ eq./kg-PP. The manufacturing process of propylene has extremely high impacts on global warming potential, fossil resource depletion (1.722 kg Oil eq./kg-PP), human toxicity (0.077 kg 1,4-DB eq./kg-PP), acidification (0.0049 kg SO_2_ eq./kg-PP), and petrochemical oxidant formation (0.0042 kg NMVOC/kg-PP). Additionally, based on the results of this present research, this study proposes possible improvements and alternative solutions such as applying advanced technologies, clean energy, and safe recycling processes in the GCC that are environmentally friendly.

## 1. Introduction

The petrochemical industry is considered one of the most rapidly growing petroleum industries that fulfill most of the world’s industrial and financial needs. By producing the largest daily volume of petrochemical products such as plastics, pharmaceuticals, fertilizers, and textile products, this industry is the leading industry for many industrial zones in the world. Furthermore, the need for these petrochemical products has grown rapidly and dominates the global chemical market. As a result, the petroleum and petrochemical industry has experienced sustained growth and remains a critical factor in the underlying economies of every industrial nation [1]. The petrochemical industry also includes the production, manufacturing, and treatment of petrochemical products, which are then provided to several other sectors. Thus, the search for manufacturing industries to produce innovative products has increased tremendously. The mechanical properties of PP are comprised of good flexural power, impact strength, rigidity, a higher strength-to-weight ratio, modulus, and thermal properties that include temperature deflection. New materials produced with PP are considered cost-effective and are used in various industries [2,3]. Thus, PP and its different blends have become the second most fundamental product category among plastic products, with revenues exceeding the US $124.01 billion by 2019. Hence, it is inevitable that demand for thermoplastics products will increase, which will likely meet the global production of PP in the near future.

PP can be recycled, which makes it ideal for use in various applications [4]. It is also used heavily in the automotive industry to manufacture parts. According to a study by Maddah [5], the international need for plastics reached 245 million tons per year, which is likely to grow significantly due to the rise in public demand. The redundant use of plastic products, and the resulting risks associated with plastics, have equally threatened both human and aquatic life and changed entire natural environments [6,7]. The PP covers more than 25% of the polymer market demand; the PP marketplace is the second-largest polymer industry worldwide [8]. The production capacity of PP varies according to global and regional market demand and is affected by numerous factors, including oil prices and new technological inventions. This results in lower PP prices worldwide, providing cheap feedstock, particularly for PP manufacturers worldwide. It has been estimated that the production cost of a barrel of feedstock is 60% less compared to 10 years ago [9].

**Figure 1:**
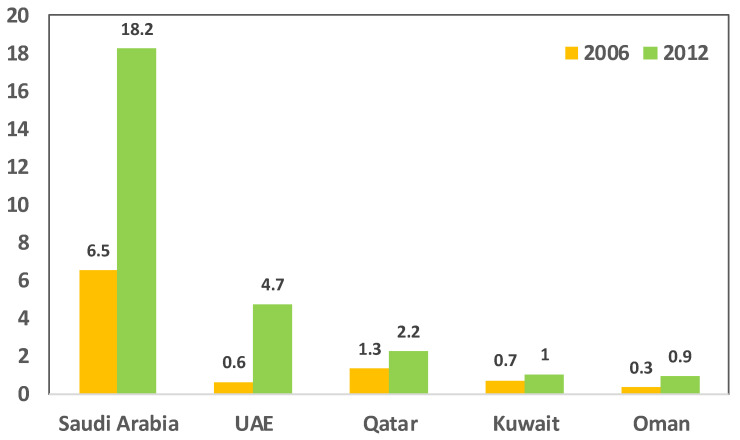
Polymers production capacity (in million tons per annum, MTPA) in the GCC region for the years 2006 up to 2016 [10].

The GCC is considered the major producer and exporter of PP to markets worldwide and is a steady global supplier [8]. This is due to the fact that the petrochemical industry is significant in the GCC due to the raw products of crude oil and natural gas within the region. The petrochemical industry in the GCC expanded significantly and stabilized using its access to one-third of the world’s oil and natural gas reserves. As a result, the industry has become a solid contributor to the region’s economic development, and it continues to flourish. Affordable energy and the availability of cheap transport and resources are the driving factors for industrial growth in this region. This has enabled regional oil and gas resources to develop the chemical industry. Figure 1 demonstrates the jump in the polymer industry and the rapid growth from 2006 up to 2016 [10].

Keeping in mind the rapidly growing consumption and demand of PP products worldwide, it has become important to optimize the PP manufacturing process, improve it, and examine the effects of manufacturing PP products on the environment. Such careful research studies aim to ensure that PP production takes the most sustainable and environment-friendly approach, rooting from the facts-driven suggestions from this assessment. Furthermore, polymers have been used to produce thermally enhanced polymers in the past few years due to good heat transfer characteristics that may replace the metallic parts that require fabrication at high temperatures (and hence more energy) [11,12]. On the other hand, the fabrication of thermally enhanced polymers is simpler, and a lower operating temperature is needed to produce a part. Thus, the environmental impacts of polymers play a vital role in conducting life cycle assessment (LCA) of thermally enhanced polymers to compare with the environmental effects of the metallic parts. The main objective of this study is to try and provide substantial information on the multiple applications of PP and to develop a context for the rapidly rising demand for PP products.

Moreover, this study focuses on understanding the industrial manufacturing process of PP by conducting an LCA study based on an existing PP production plant located in the GCC to quantify the environmental impacts of each stage of the PP manufacturing process. The LCA results for the manufacturing process of one ton of PP pellets based on the plant located in the GCC region should deliver a clear overview for the decision-makers in the petrochemical industries sector and the customers of polymer producers in the GCC to be aware of the environmental impacts and how to improve the PP production technology. This LCA study aims to encapsulate the differences and uses among the PP grades while also providing a proper and complete assessment of the environmental impacts of PP manufacturing. It also draws conclusions based on the study regarding the PP industry with its increasing demands and applications. Therefore, researchers can conduct LCA studies for applications of PP based on this LCA study. Furthermore, the environmental impacts of the manufacturing process of PP are weakly quantified or understood in the GCC due to the confidentiality of the PP plant data in the petrochemical sector.

## 2. Literature Review

With the rise in demand for manufacturing plastic products, developing a mechanism to protect against environmental degradation has remained a concern. Thus, there is still a need to analyze what is available in the market and come up with better strategies in the manufacturing process in order to reduce pollution [13]. This is possible through research focused on developing better combinations that may result in a decrease in pollution, although current research has focused on the impacts of global warming and depletion of fossil fuels due to the manufacturing of products made with plastic and petroleum-based products [14]. The research in question concerned how PP manufacturing can be improved to make it more efficient and less harmful to the environment, as it is among the most useful polymers in plastics manufacturing. To understand the challenges associated with polymers manufacturing, it is important to analyze the pollution and environmental degradation levels of polymers.

PP is an addition-polymer whose manufacturing process involves the combination of several monomers. This implies there is still a research gap regarding whether other methods could be applied in manufacturing to make this product more effective [15]. In terms of environmental damage, the risks associated with plastic production have created a need for precise ways to decrease the extent of environmental burdens associated with PP manufacturing, especially as the demand for them increases [16]. Therefore, Grünberg studied the important methodologies used to improve the efficiency of the manufacturing process, beginning with the industrial age [17]. Furthermore, since PP can be recycled, this strategy can also be explored as a mechanism to reduce pollution. The high pollution level within the environment is a clear indicator that better methods have not been identified to handle the recycling process. Thus, this represents another gap that needs to be addressed, although the recycling aspect is not considered in this work.

Greener production refers to using environmentally friendly principles, strategies, and technologies to achieve environmentally sustainable production that substitutes manufacturing materials with safer and non-toxic materials [18,19,20]. This is possible by developing better mechanisms that focus on the manufacturing processes of PP through the implementation of the LCA tool [21]. The LCA provides environmental management by promoting an eco-friendly manufacturing approach. LCA is one of the frequently used key tools to evaluate the usage of power and energy, water, and other resources, including the emission of toxic gases into the air, removal of industrial wastes, and other harmful effects as a result of several industrial manufacturing processes [22,23,24]. Therefore, the LCA tool would be a practical solution for reducing environmental pollution, especially since plastic products are highly demanded, and the safe recycling of plastics should be addressed. The LCA tool was beneficial for conducting a comparative analysis for single-use plastic products and reusable plastic products. For instance, a previous LCA study has been conducted in California State specifically for plastic bags that were made from different types of polymers in order to examine the environmental impacts and the durability of the reusable plastic bags comparing to single-use bags [25]. It was proven that the environmental impacts of reusable polypropylene bags are lower than single-use plastic and paper bags. Furthermore, the equivalent carbon emissions for PP and polyethylene (PE) were estimated to be 1.34 and 1.48 kg CO_2_ eq. per unit kg of polymer, correspondingly.

Ingarao et al. [26] carried out LCA of food packaging materials (i.e., tin steel, PP, and glass). They found that the LCA of PP exhibits lower global warming potential and energy requirements than other materials. The Gulf Petrochemicals and Chemicals Association (GPCA) conducted LCA for PP and high-density polyethylene (HDPE) in the GCC region [27]. They estimated the carbon emissions associated with the PP production as 1.95 kg CO_2_ eq. per unit kg of PP. However, this study is particular and detailed compared to the GPCA study since it was conducted based on an existing PP plant located in a certain country in the GCC region, which remains anonymous due to confidentiality reasons. Narita et al. [28] analyzed the life cycle impact of PP production in Japan using the cradle to gate approach, and they found the equivalent carbon emissions of 1.4 kg CO_2_ eq. per unit kg of PP. Overall, the various studies conducted on PP and its associated products prove that even though there is a growing interest in the use of plastics. Therefore, future research on this subject must focus on efficient processes, use of renewables for required heat and electricity, better recycling methods, and developing combinations that would make PP less polluting, as it is used as a raw material for manufacturing many kinds of commercial products.

## 3. Methods

In this study, the LCA approach is followed to assess the environmental impacts associated with the PP manufacturing process. This approach provides the opportunity to determine the effects of each specific stage of the manufacturing process of PP, which consists of four main steps.

### 3.1. Description of the Analyzed PP Plant

**Figure 2:**
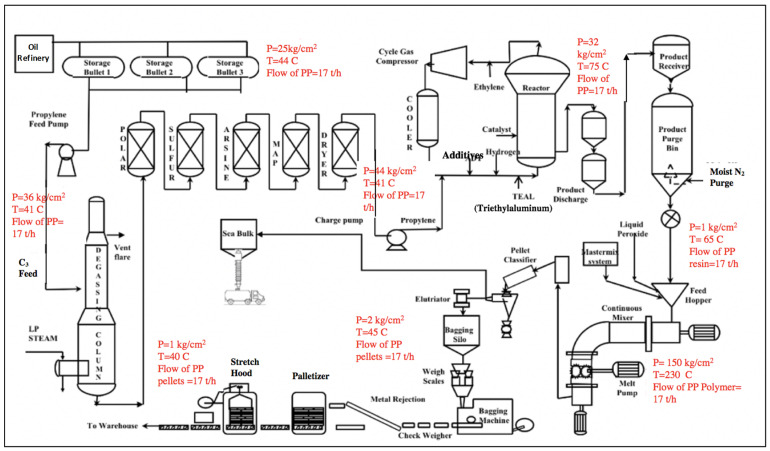
Process flow diagram of the PP manufacturing process obtained from an existing plant in the GCC.

Many PP plants are operating around the world. Each plant uses a special process technology for PP production. Moreover, each PP plant produces different types of PP grades based on market demand. This LCA study is conducted to identify the associated environmental impacts of an existing PP plant in the GCC. This particular plant uses UNIPOL PP processing technology, a gas-phase technology that occurs in a fluidized bed reactor system. There are three main grades of PP produced in this particular plant. The first is the crack grade (HT-251), which is mainly used for fiber applications. The second grade is the raffia grade (HT-031), which is mainly used for tape applications. Finally, biaxial-oriented PP (BOPP or HF-029) is used for film applications. These three PP grades differ in the melt flow rate, as well as in their applications. To switch from one grade to another, a fixed technology process is used, but the manipulated variable is the percentage of Xylene. Figure 2 demonstrates the schematic flow diagram of the PP manufacturing process that has been implemented in the PP plant located in the GCC.

In this particular PP plant, the manufacturing process of PP consists of four main areas or stages which are as follows: the utility and purification area, the reaction area, the pelleting area, and finally the packaging area. The LCA approach used in this PP plant will measure the environmental implications for the whole manufacturing process. PP granular pellets specifically for the raffia grade are produced from liquid propylene (C_3_H_6_). As illustrated in Figure 2, the raw material for propylene is supplied from two different sources. The first one comes from another petroleum refinery. In contrast, the second source comes from an ethylene unit where the propylene is produced as a by-product and pumped to another unit to be purified before being stored. Generally speaking, the propylene is considered a raw material of this process, is received from those two propene sources, and is then stored in a storage system that consists of three main propylene pressure storage bullets, which are labeled in Figure 2 as storage bullet 1, 2, and 3. Each storage bullet has a capacity of 100 tons. Afterward, the liquid propylene is fed into the degassing column through a charging pump to eliminate undesired gases such as CO, CO_2_, and O_2_. This process occurs due to volatility due to differences in the boiling points, as heavy gases will exit from the bottom of the column and light gases will exit from the top of the degassing column. Next, the propylene will pass by the purification unit to purify the propylene before entering the reaction unit. After purifying the liquid propylene, it passes through the reaction area where the main purpose is to convert the liquid propylene into powder resin PP. At this specific stage, the grade type can be decided as it is the area where the catalyst is fed at a certain percentage to produce a particular PP grade, which is decided before running the plant. Next, the PP resin is fed into the pelleting area, where the main objective is to convert the powder PP resin into pellets of a specific size. Finally, the PP pellets are transported using a conveying system to the packaging area.

According to the data collected from the PP manufacturing plant located in the GCC, the original plant design capacity is 100 kilotons annually. It currently operates at 145–150 kilotons annually. The annual production of PP pellets is estimated to be 146,880 tons.

### 3.2. LCA Framework

LCA is a scientific methodology used for quantifying the environmental burdens associated with a process or product. PP products are high in demand and are widely used polymers for several applications around the globe. For this reason, it is crucial to assess the environmental impacts of manufacturing PP, especially when the PP production and market demand are increasing rapidly day by day. The LCA of commonly used products will help measure the environmental burdens that occur during the production of PP, which are based on an existing plant located in the GCC. This LCA study was conducted for an existing PP plant located in the GCC, but due to confidentiality, the name and location of the PP plant remain anonymous. Furthermore, this LCA study follows the standard LCA framework, which consists of four main steps named as follows: goal and scope, life cycle inventory (LCI), life cycle impact assessment (LCIA), and the LCA interpretation phase, which were formalized and defined by the International Organization for Standardization (ISO), 14,040 and 14,044 [29,30].

#### 3.2.1. Goal and Scope of the LCA

This particular stage of the LCA study is extremely significant. The main purpose of this study is defined to make it easier for reviewers or the audience to understand the benefits of conducting this LCA study. The main goal of this LCA study is to quantify the environmental impacts associated with the manufacturing process of PP, based on a case study that has been implemented for an existing PP plant located in the GCC. Since an LCA study is used for the manufacturing process of PP production, the functional unit is defined in terms of the system’s output, which is determined to be one ton of PP pellets of raffia grade. Generally speaking, a functional unit is considered the basis upon which this LCA study is conducted. This LCA study will be beneficial for future researchers who wish to conduct LCA studies for products that are made of PP since this study focuses on measuring the environmental impacts associated with the manufacturing process of 1 ton of PP pellets according to data obtained from an existing PP plant located in the GCC. The PP pellets are used as a raw material for many other applications.

**Figure 3:**
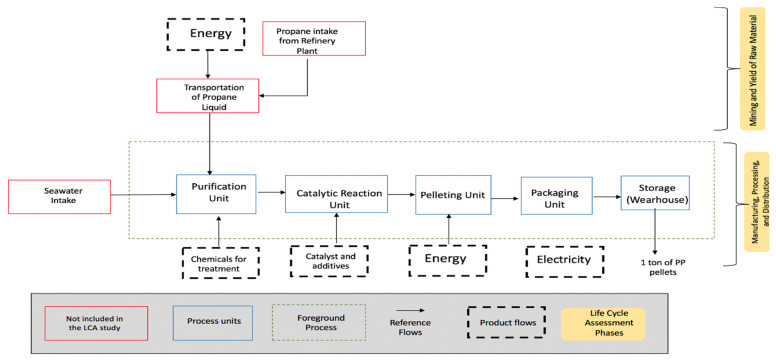
System boundaries for each unit in the PP manufacturing plant considered in this LCA study. The life cycle phases have been highlighted, while the construction of this plant, production of raw material, usage of PP, and the end-life scenarios for PP have been excluded from this LCA study.

For modeling, the GaBi software, a LCA tool was used to model or construct the LCA flow chart for this particular PP manufacturing process [31]. To evaluate the environmental burden, the Ecoinvent database was used. Finally, for the impact assessment method, the Recipe 2008 handbook was used. Figure 3 demonstrates the system boundaries for this LCA study, which shows the whole manufacturing process for one-ton PP pellets, set as the functional unit for this LCA study. The system boundary presents the life cycle phases that have been considered for this particular LCA study and which are obtained from raw material that is fed to the PP plant to produce one ton of PP. It excludes the construction of the plant and the end-life or usage of PP. Moreover, the flowchart of the LCA for the PP manufacturing plant was generated using GaBi software, version 4.2, Leinfelden-Echterdingen, Germany [31].

#### 3.2.2. Life Cycle Inventory Analysis

At the life cycle inventory analysis (LCI) phase, all of the data regarding the production of one ton of PP pellets were collected from an existing plant in the GCC, based on the input and output data of the plant. At this stage of the LCA study, the energy and raw material requirements and the atmospheric emissions are quantified for the results of the LCI analysis. Most of the data analysis used in this section is obtained from a technical advisor working at the PP plant. The GaBi database is explicitly used for the energy data input for the GCC. The chemical formula of the catalyst is extremely confidential and could not be provided due to confidentiality from the manufacturer. However, the catalyst used in the PP plant is called Shell High Activity Catalyst 320 (SHAC 320).

Moreover, the PP manufacturing process selected for this study has a high degree of influence on the results of the LCI analysis, where the basis of this process had a flow rate of 17 tons of PP pellets per hour. Eventually, all of the results are converted to meet the actual functional unit, namely one ton of PP pellets (specifically the raffia grade). Technically speaking, the process of producing other PP grades doesn’t differ in the technology applied for the manufacturing process studied in this LCA study. However, some factors can be manipulated in the plant to switch from producing one grade to another based on the market demand since each PP grade is produced for distinct applications.

#### 3.2.3. Life Cycle Impact Assessment

The life cycle impact assessment (LCIA) is the phase in which the real data is obtained from the existing PP plant and used to analyze the environmental impacts of the PP manufacturing process. Table 1 shows the impacts of the PP production process, which are examined after inventory data collection. The following impact assessment methodology came from Recipe 2008, which contains several impact categories [32]. Some of these are selected for study in this research, where the process of choosing the impact categories have been carried based on three major geographical scalings which are as follows: global, regional, and local effects. Hence, Table 1 illustrates the impact categories selected for this LCA study, which shows the highest variations for the ease of understanding and mitigating. Furthermore, the impact categories for this LCA study are selected based on the most available impact categories in the literature and public reports that are related to PP for better comparison purposes with previous studies. Moreover, this LCA study is conducted based on the ISO14040 and 14044:2006; the LCIA categories are selected to cover endpoint categories which are: human health, environment, and issues resulting from the consumption of natural resources, wherein the LCIA these endpoint subcategories are refined into mid-point categories as demonstrated in Table 1.

#### 3.2.4. LCA Interpretation

The production of PP pellets in the analyzed plant consisted of several processes, which are as follows: feed and purification process, reaction processes, pelleting processes, and packaging. The overall environmental loads have been determined for the entire manufacturing process of PP.

## 4. Results and Discussion

### 4.1. LCA Results

The PP manufacturing process releases numerous pollutants into the environment. This is proven throughout the LCA study, which is conducted for this particular process using GaBi software. Some common pollutants are selected in this study based upon their impact categories, which are listed in Table 1. The gross per ton of PP and kg of PP LCA impacts, are presented in Table 2, correspondingly. The global warming potential (GWP) is calculated as the equivalent mass of carbon emissions released to the environment. The PP manufacturing plant located at GCC region contributes to approximately 27 tons of equivalent CO_2_ emissions annually that is certain to have both short and long-term impacts on the surrounding environment and human health. For the functional unit, i.e., 1 ton of PP, the estimated equivalent carbon emissions are 1586 kg of CO_2_, which means the weight of associated carbon emissions is more than the weight of the product. Therefore, it is essential to implement modifications or apply new technologies that are more environmentally friendly to cut down on CO_2_ emissions and other undesired emissions. For example, the heat and electrical energy required can be furnished by means of renewable sources (cleaner energy mix) that may lower the overall GWP impact and the production cost as the power from renewables have achieved lower prices than the fossil-based power [34,35]. In addition, CO_2_ from the carbon capture can be utilized for polymers production [36].

The terrestrial acidification (TA) implies the decline in soil fertility due to the accumulation of nitrogen and sulfur-based acidic forms such as NO_x_, SO_2,_ and NH_3_. The total impact is evaluated in terms of an equivalent mass of SO_2_. The production of PP pellets causes 84.92 kg of SO_2_ annually for total production and 4.99 kg of SO_2_ per ton of PP pellets. The petrochemical oxidant formation (POF) is estimated by the total emissions of non-methane volatile organic compounds (NMVOC), including formaldehyde, ethanol, benzene, cyclohexane, acetone, etc. The presence of NMVOC raises the concertation tropospheric ozone and aerosols, and can significantly affect the human health such as cardiovascular and respiratory diseases.

**Figure 4:**
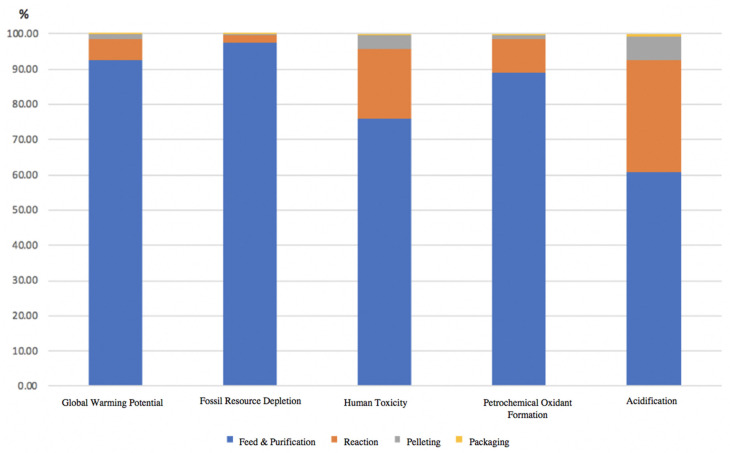
An overview for the impact assessment categories results for annual production of PP, representing the results for each process in the PP manufacturing plant, including the feed and purification phase.

Furthermore, some of the NMVOC substances are carcinogenic. One ton of PP production emitted 4.24 kg of NMVOC as the PP is the derivative of petrochemical product, so volatile organic compounds emissions during production are reasonable. The fossil resource depletion (FD) indicates fossil-based resource consumption during a particular process and is calculated as kg of oil equivalent. The PP pellets manufacturing required 1.72 tons of oil. As the PP is a petrochemical product, so the usage of fossil resources is understandable. However, if it is possible to use alternatives such as biofuels, this impact may be reduced. The human toxicity (HT) impact determines how much a process can harm human health and is measured as the equivalent weight of 1, 4-dichlorobenzene (DB) equivalents (kg 1,4-DB eq.). The HT for one ton of PP pellets manufacturing is found to be 77 kg 1,4-DB eq. Both HT and POF represent major impacts related to human health. The analyzed areas have been divided into four sub-processes for the ease of understanding, and the relative contribution for each phase is demonstrated in Figure 4. It can be seen that the largest percentage of pollutants are released from the feed and purification phase, where during that phase, the propylene liquid being purified and fed to the examined PP plant. Each impact category represents the different phases of the PP manufacturing process, where each process had different emissions. For instance, the largest number of emissions are coming from the feed and purification phase, followed by the reaction phase.

**Figure 5:**
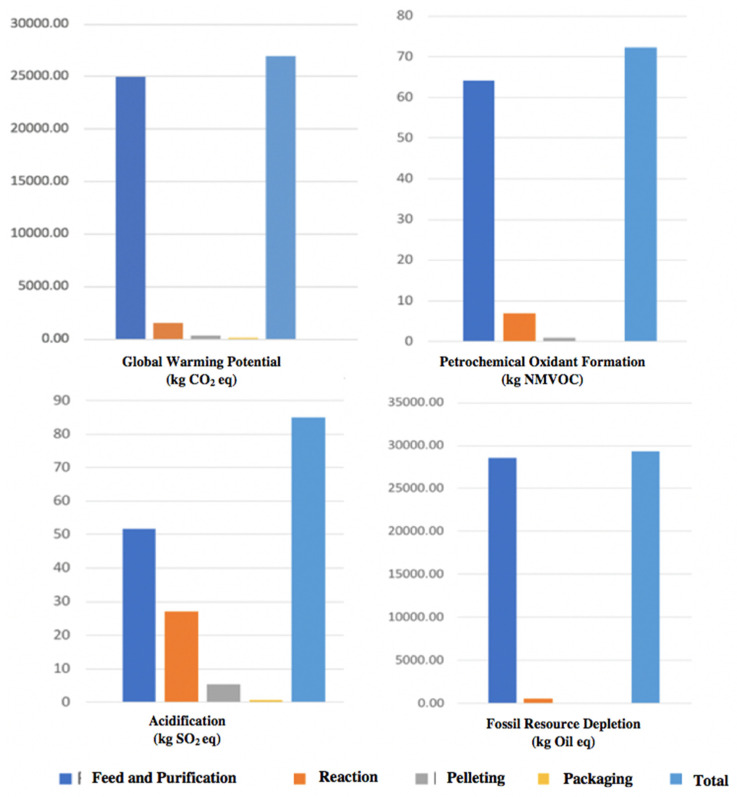
An overview of the impact categories results for each phase of the PP manufacturing process, where each category is represented separately based on the annual production of PP pellets.

According to Figure 5, the feed and purification phase has the highest results for all of the impact categories selected in this LCA study as compared to other PP manufacturing phases. The reaction phase represented the second-largest emissions. Furthermore, the pelleting and packaging processes have negligible contributions in GWP, POF, and FD compared to total LCA impacts. However, pelleting and packaging processes have some impact in terrestrial acidification. Table 3 represents a quantitative overview for the global warming potential impact assessment results, measured for each phase of the PP manufacturing plant for the annual production for 1 ton of PP pellets and 1 kg of PP pellets. The amount of gross CO_2_ emissions is estimated to be 1.58 kg CO_2_ per kg of PP pellets and 1586.4 kg CO_2_ per 1 ton of PP pellets.

### 4.2. Results Comparison with Previous LCA Studies

The gross carbon dioxide emission for the PP manufacturing plant located in the GCC is compared with the results of other LCA studies that have been conducted previously in the literature, and with the values available in the Gabi database for US and Germany. There are differences between this LCA-based study and the previous studies regarding process inputs, energy and process efficiency, methodology, and datasets certainty. The value of gross carbon dioxide emissions obtained from this study are compared to the results of earlier studies as listed in Table 4. The variations in the results are mainly due to the difference in data sets used, which involves energy and process efficiency, energy requirements, type of material, and process type, etc. Moreover, the calculation method and the modeling software may differ from one study to another. For instance, this LCA study is very specific and detailed since it is applied for one specific PP plant located in one of the GCC countries. However, the study funded by GPCA was conducted based on data sets collected from seven petrochemical plants located in different GCC countries (four from Saudi Arabia, one from Oman, Kuwait, and UAE, respectively). During the modeling and calculation, vertical averages were calculated using the vertical averaging method to get the best outcome [27]. As the fuel type, energy mix, production capacity, plant age, energy and process efficiency, and process type may differ from plant to plant, which can augment the average carbon emissions values as in GPCA study. The carbon emissions evaluated in this study are very closer to the studies carried out for California, US, Germany, and Japan [25,28]. However, the carbon emissions are slightly higher due to different energy mix/energy sources in the US and Japan. As per the Gabi database for the US, the carbon emissions are higher than the GPCA study that shows the LCA results strongly depend on the plant particulars. In addition, the study conducted by GPCA [27] and Gabi database for the US reflects higher carbon emissions that are due to the reasons explained above. However, the GWP value from the present work aligns within the range available in the literature.

### 4.3. Data and Results Quality and Uncertainty

For the GCC region, most previously conducted LCA studies have been for products that are made from PP or other polymers, but very few studies have been conducted for a PP manufacturing plant. Thus, this particular study may have some uncertainties for several reasons, which are demonstrated below:

- The data that was collected from the existing PP plant, and is confidential (such as energy mix, details of the processes, name of the plant cannot be furnished in this work).
- Other data not provided from the PP plant were instead obtained from GaBi software and applied to other regions.

## 5. Conclusions and Outlook

PP is a heavily utilized material that is the backbone of countless significant industries, and products made with PP are used globally. This has resulted in the rapid and extraordinary growth of plastic production, surpassing most other synthetic materials. Thus, the global production of PP is increasing tremendously every year to meet the growing demand. It has become necessary to understand the chemical composition of PP, its several grades, and its applications. Therefore, this research is conducted to thoroughly examine the impact of the PP manufacturing process on the ecosystem. This is carried out using LCA in Gabi software. Through this LCA process, this study aspires to observe and measure the environmental burdens associated with the manufacturing of PP. It also aims to implement further technologies to reduce the environmental impacts of the PP manufacturing processes. Some of the key findings of our work are as follows:

- The results indicate 1.58 kg of carbon emissions and 1.72 kg of oil depletion for each kilogram of PP pellet production.
- The feed and purification phase has the highest contribution environmental impacts followed by reaction process. Furthermore, the pelleting and packaging processes have negligible contributions in GWP, POF, and FD compared to the total environmental impacts of the PP plant. However, pelleting and packaging processes have a smaller impact on terrestrial acidification.
- The GPCA study showed 1.95 kg of equivalent carbon emissions for the production of 1 kg of PP pellets. This value represents the averaging of PP manufacturing plants in the GCC region and is more than the present study. The variation is mainly due to the difference in data sets used as the study by GPCA was based on data sets collected from seven petrochemical plants located in different GCC countries. The fuel type, energy mix, production capacity, plant age, energy and process efficiency, and process type may differ from plant to plant, increasing the average carbon emissions values
- The GWP in this work is compared with values available in the literature, and it is found that the present work aligns within the range available in the literature. These results guide the petrochemical industrial sector to shift the production technology, especially the feed purification and reaction phases since they have the highest impact results to overcome undesired environmental impacts such as greenhouse gas emissions.

There remains a gap that hinders effective waste management, which would offer a lasting solution for environmental impacts. This does not eliminate the fact that PP is affordable and has high flexural strength due to its semi-crystalline nature, with a low coefficient of friction and better chemical resistance to many bases and acids. Therefore, future research on this subject must focus on better recycling methods and developing combinations that would make PP less polluting. Moreover, the PP production process should be more sustainable and environmentally friendly, as it is used as a raw material for manufacturing many kinds of commercial products.

## Figures and Tables

**Table 1:** An overview of the mid-point impact categories selected from the Recipe handbook 2008 [33].

Impact Categories	Abbreviations	Units	Scale
Terrestrial Acidification	TA	kg SO_2_ eq.	Regional
Petrochemical Oxidant formation	POF	kg NMVOC	Local
Human Toxicity	HT	kg 1,4-DB eq.	Local
Fossil Resource Depletion	FD	kg oil eq.	Regional
Global Warming Potential	GWP	kg CO_2_ eq.	Global

**Table 2:** Detailed results for the number of emissions released into the atmosphere during the PP manufacturing process (based on functional unit = 1 ton of PP pellets).

Impact Categories	Units	Emissions per Annual Production (146,880 Tons of PP Pellets/yr)	Emissions per 1 ton of PP Pellets	Emissions per 1 kg of PP Pellets
TA	kg SO_2_ eq.	84.92	4.99	0.0049
GWP	kg CO_2_ eq.	26,968.80	1586.35	1.5863
POF	kg NMVOC	72.20	4.24	0.0042
FD	kg Oil eq.	29,289.40	1722.90	1.7222
HT	kg 1,4-DB eq.	1309.16	77.00	0.0770

**Table 3:** An overview of the global warming potential impact category results, which were measured for each process of the PP manufacturing plant.

GWP	Feed & Purification	Reaction	Pelleting	Packaging	Total (kg CO_2_ eq.)
For Annual Production	25,000.000	1600.000	326.000	42.800	26,968.800
For 1 ton of PP pellets	1470.588	94.118	19.176	2.518	1586.400
For 1 kg of PP pellets	1.471	0.094	0.019	0.003	1.586

**Table 4:** A relative comparison of the gross carbon dioxide emission results per 1 kg of PP between this LCA study and two previous LCA studies.

Resource	Location	Equivalent CO_2_ Emission per 1 kg of PP
Life cycle inventory analysis of CO_2_ emissions: Manufacturing commodity plastics in Japan [28]	Japan	1.4 kg CO_2_ eq.
This LCA study	One of the GCC countries	**1.58 kg CO_2_ eq.**
GPCA study [27]	The entire GCC region	1.95 kg CO_2_ eq.
LCA of single use and reusable plastics bags [25]	California, US	1.34 kg CO_2_ eq.
Gabi database [31]	US	2.41 kg CO_2_ eq.
Gabi database [31]	Germany	1.64 kg CO_2_ eq.

## Data Availability

Restrictions apply to the availability of these data. Data was obtained from the commercial PP manufacturing plant and are available with the permission of the company.
